# Ovarian Fragmentation and AKT Stimulation for Expansion of Fertile Lifespan

**DOI:** 10.3389/frph.2021.636771

**Published:** 2021-03-02

**Authors:** Kim Cat Tuyen Vo, Kazuhiro Kawamura

**Affiliations:** Department of Obstetrics and Gynecology, Advanced Reproduction Research Center, International University of Health and Welfare, Narita, Japan

**Keywords:** Akt stimulation, diminished ovarian reserve, *in vitro* activation, ovarian fragmentation, premature ovarian insufficiency

## Abstract

Since the first baby was born after *in vitro* fertilization, the female infertility treatment has been well-developed, yielding successful outcomes. However, successful pregnancies for patients with premature ovarian insufficiency and diminished ovarian reserve are still difficult and diverse therapies have been suggested to improve the chances to have their genetically linked offspring. Recent studies demonstrated that the activation Akt pathway by using a phosphatase and tensin homolog enzyme inhibitor and a phosphatidylinositol-3 kinase stimulator can activate dormant primordial follicles in both mice and human ovaries. Subsequent researches suggested that the disruption of Hippo signaling pathway by ovarian fragmentation increased the expression of downstream growth factors and secondary follicle growth. Based on the combination of ovarian fragmentation and Akt stimulation, the *in vitro* activation (IVA) approach has resulted in successful follicle growth and live births in premature ovarian insufficiency patients. The approach with disruption of Hippo signaling only was also shown to be effective for treating poor ovarian responders with diminishing ovarian reserve, including advanced age women and cancer patients undergoing sterilizing treatments. This review aims to summarize the effectiveness of ovarian fragmentation and Akt stimulation on follicle growth and the potential of IVA in extending female fertile lifespan.

## Introduction

On July 25, 1978, the first baby was born after conception by *in vitro* fertilization (IVF), establishing a new medical approach, giving the chance to achieve parenthood to more than 10 million couples. Over the past decades, there have been remarkable advances in assisted reproductive technologies, resulting in a higher successful live birth rate (1). However, the treatment outcomes for patients with ovarian dysfunction including premature ovarian insufficiency (POI) and poor ovarian response with diminishing ovarian reserve (POR-DOR) have limited success. Oocyte donation or adoption which cannot help them to have their genetic children are often their only options. Recently, there is a significant rise in the mean age of marriages, leading to a higher rate of advanced age women seeking infertility treatment (2, 3). Since ovarian dysfunction cannot be treated by conventional gonadotropin stimulation, new therapeutic interventions are needed to stimulate follicle growth.

Among the pathways modulating early folliculogenesis, the phosphoinositide 3-kinase (PI3K)/Akt signaling pathway plays a crucial role in the activation of primordial follicles (4–6). Subsequent experiments suggested that the Hippo signaling pathway is also important in the development of follicles (7–9). The disruption of the Hippo signaling pathway by ovarian fragmentation was demonstrated to increase the actin polymerization, leading to the nuclear translocation of Yes-associated protein (YAP), the increased downstream cysteine-rich 61, connective tissue growth factor, nephroblastoma overexpressed (CCN) growth factors, and baculoviral IAP repeat containing (BIRC) apoptosis inhibitors (10), resulting in follicle growth enhancement (5, 7–9). The combination of ovarian fragmentation and incubation in the presence of Akt-stimulators developed a new therapy named *in vitro* activation (IVA) for treating POI patients by our group. Following the IVA treatment, healthy live births have been reported (7, 11, 12). We also developed drug-free IVA, a simplified approach of IVA, to treat patients with POI at early stage and for POR-DOR patients (13–15). In addition, we demonstrated that laparoscopic ovarian incision could activate the follicles *in vivo* and was a potential therapy for patients with resistant ovary syndrome (ROS) (16).

These results revealed that ovarian fragmentation and Akt stimulation could improve the infertility treatment outcomes for different categories of ovarian dysfunction. This review summarized the knowledge of ovarian fragmentation and Akt stimulation effectiveness on follicle growth and their potential in female fertility expansion.

## The Important Role of AKT Simulation in Primordial Follicle Activation

The mammalian ovary is a complex organ containing follicles as basic functional units (17). To develop preovulatory follicles containing mature oocytes, a number of small primordial follicles are periodically activated from the pool of primordial follicles to undergo folliculogenesis. Although the mechanisms of selection and activation of dormant primordial follicles are yet not fully clarified, recent studies suggested several important intracellular signaling mechanisms to activate dormant primordial follicles (18). Among these pathways, the PI3K/Akt/forkhead box O3 (FOXO3) pathway is the principal one in primordial follicle activation (8, 18, 19). This pathway is shown to be activated by granulosa cell-produced Kit ligand (KL) (18). Meanwhile, phosphatase and tensin homolog deleted on chromosome 10 (PTEN) and tuberin/tuberous sclerosis complex (TSC1/2) negatively regulate this pathway (19). In addition, anti-Müllerian hormone (AMH) was described to have inhibitory action to the primordial follicles as it inhibits the KL (20–23). However, AMH action of follicle growth varies by species and follicular stages (24, 25).

**Figure 1:**
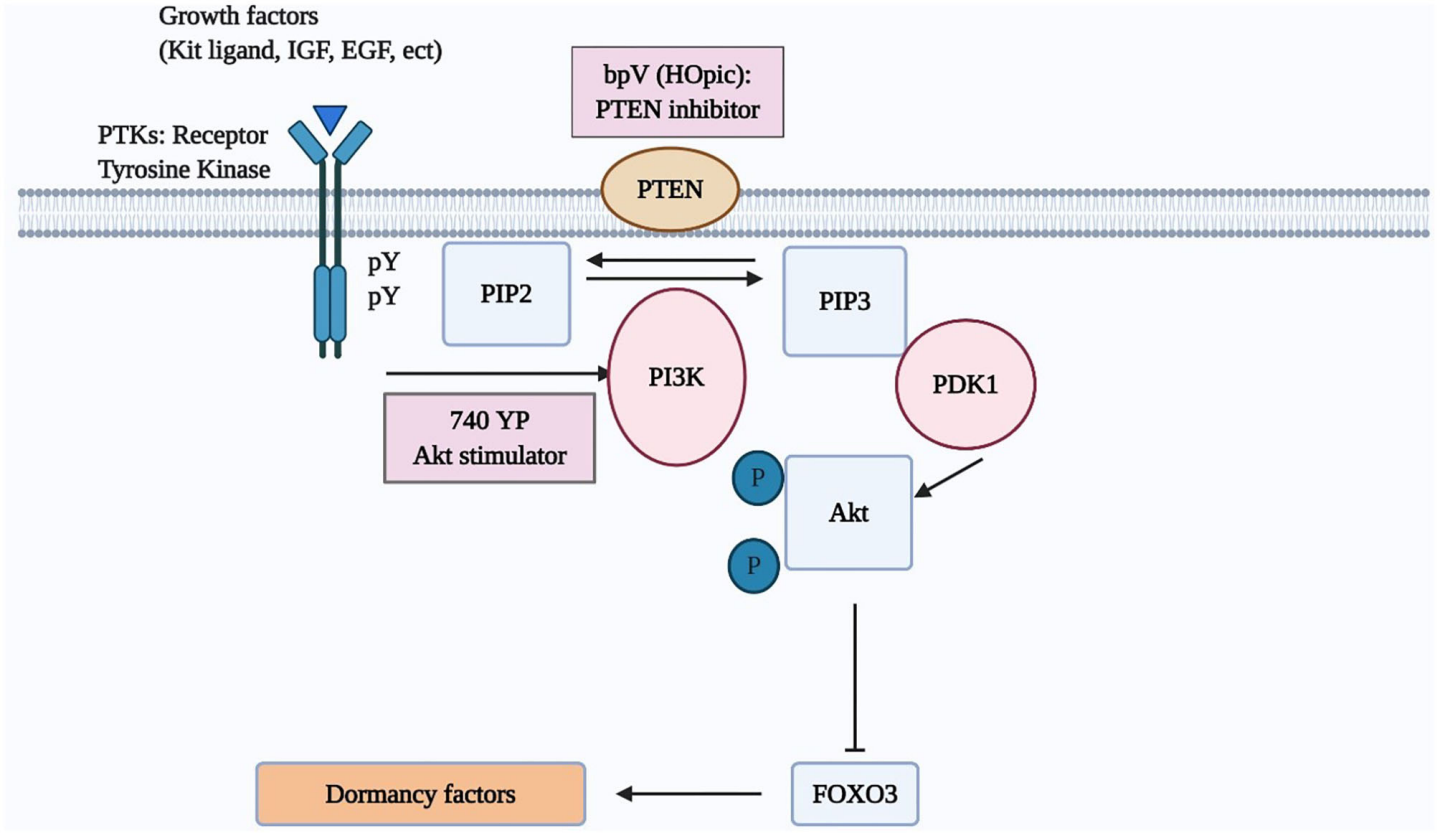
The PI3K/AKT/FOXO3 pathway in oocytes regulates primordial follicle activation. Mouse models were used to investigate the regulation of primordial follicle dormancy. The FOXO3 gene in primordial oocytes serves as a break to prevent the initiation of follicle growth. Activation of upstream RTKs by their cognate ligands (kit ligand, IGF-1, EGF, platelet-derived growth factor [PDGF], VEGF, etc) stimulates the autophosphorylation of intracellular regions of these receptors. Activated receptors then stimulate PI3K activity, leading to increases in PIP3 levels and AKT stimulation. Activated AKT then migrates to the cell nucleus and suppresses FOXO3 actions to promote primordial follicle growth.

Once Kit ligand binds its cognate tyrosine kinase receptor (c-kit), the phosphorylation of the intracellular region of c-kit enhances PI3K activity capable of transforming phosphatidylinositol-4,5-bisphosphate (PIP2) into phosphatidylinositol-3,4,5-triphosphate (PIP3). Subsequently, PIP3 stimulates phosphatidylinositol-dependent kinase 1 (PDK1), followed by the increased phosphorylation of Akt and nuclear exclusion of the transcriptional factor, FOXO3 (4, 19, 26). FOXO3 suppresses the activation of primordial follicles (18, 27) (Figure 1). Besides, Akt also stimulates cell growth through inactivation of TSC2, which is achieved by phosphorylating TSC2. The mammalian target of rapamycin (mTOR), the downstream of TSC2, regulates the tissue proliferation (18).

Several studies using the mouse model highlighted the role of the PI3K/Akt/FOXO3 pathway in activating the primordial follicles. The incubation of 3-day aged mouse ovaries *in vitro* with bpV (pic) (a PTEN inhibitor) with/without 740 YP (a PI3K activator) followed by the transplantation of paired ovaries (treated and untreated) under separate sides of the kidney capsule was presented to activate dormant follicles. After incubating for 48 h, more than half of oocytes in primordial follicles exhibited Foxo3 export. There was also an increased staining of anti-Mullerian hormone (AMH), suggesting the growth of early follicles (28). After the transplantation to hosts and stimulation with gonadotropins, increases in ovarian sizes and the number of antral follicles in the treated group were evident as compared with paired control, resulting in the delivery of healthy progeny (28). Besides, mutant mice with specific deletion of PTEN was demonstrated to increase granulosa cell proliferation, and ovulatory efficacy as well as to decrease follicle atresia (29). Other studies presented that the deletion of PTEN, TSCI/2, or FOXO3 resulted in an extensive and precocious activation of primordial follicles (30–32). In another work, mice's ovaries cultured with a different PTEN inhibitor, bpV (HOpic) alone for 24 h had a higher number of follicles at preovulatory stage and slightly higher numbers of pups compared to the controls (33). A recent study represented that a long-term bpV (HOpic) treatment alone for 6 days could promote the primordial follicle activation in bovine ovaries without applying the air-liquid interface cell culture, whereas the bpV (HOpic) treatment affected negatively on the DNA structure and its repair competence (34).

In humans, the incubation of the ovarian tissue with a PTEN inhibitor and/or a PI3K activator was reported to activate primordial follicles (7, 28, 35). After the long-term incubation with 1 μM bpV (HOpic) for 6 days without applying the air-liquid interface cell culture, the number of growing follicles in the bpV (HOpic) exposed group increased remarkably. However, the survival rate of secondary follicles from the control group was significantly higher compared to one from the bpV (HOpic) exposed group (59 vs. 27%) (36). Moreover, another work revealed that a concentration of bpV (pic) as high as 100 μM caused an extensive deterioration to follicles (37). It is consistent with a preliminary experiment in the aforementioned study showing that high doses of bpV (HOpic) (10 and 100 μM) were associated with follicular deformity (36). Meanwhile, in a subsequent experiment, incubation with 100 μM bpV (pic) alone for 25 h was showed to stimulate the follicle growth suggested by a higher percentage of growing follicles in the bpV (pic) treated group. The quantitative TUNEL assay demonstrated that the follicular viability between bpV (pic) treated and control group was not significantly different (35). As proposed in these studies, the difference in employed PTEN inhibitor, the duration of culture and its procedure may induce different outcomes (35–37).

## Induction of Follicle Growth by Fragmentation of Ovarian Cortical Tissues

The Hippo signaling pathway, initially identified in *Drosophila melanogaster*, plays a critical role in mechanotransduction and regulates mammalian organ size (38–40). It is modulated by a network of upstream components involved in cell adhesion, shape, and polarity (41). One of these components is actin, a multifunctional protein that forms microfilaments maintaining important cellular processes. The polymerization of globular actin (G-actin) to the filamentous form (F-actin) in the stress fiber has been shown to disrupt the Hippo signaling (42). The Hippo signaling kinase cascade phosphorylates the transcriptional coactivators YAP to promote its cytoplasmic localization and degradation. The disruption of Hippo signaling pathway decreases phosphorylation of YAP, thus increasing nuclear YAP levels (43). Subsequently, increased nuclear YAP interacts with transcription enhancer factor (TEF) to induce transiently the expression of CCN growth factors and BIRC apoptosis inhibitors that have positive effects on cell growth, survival, and proliferation (10, 38, 43).

**Figure 2:**
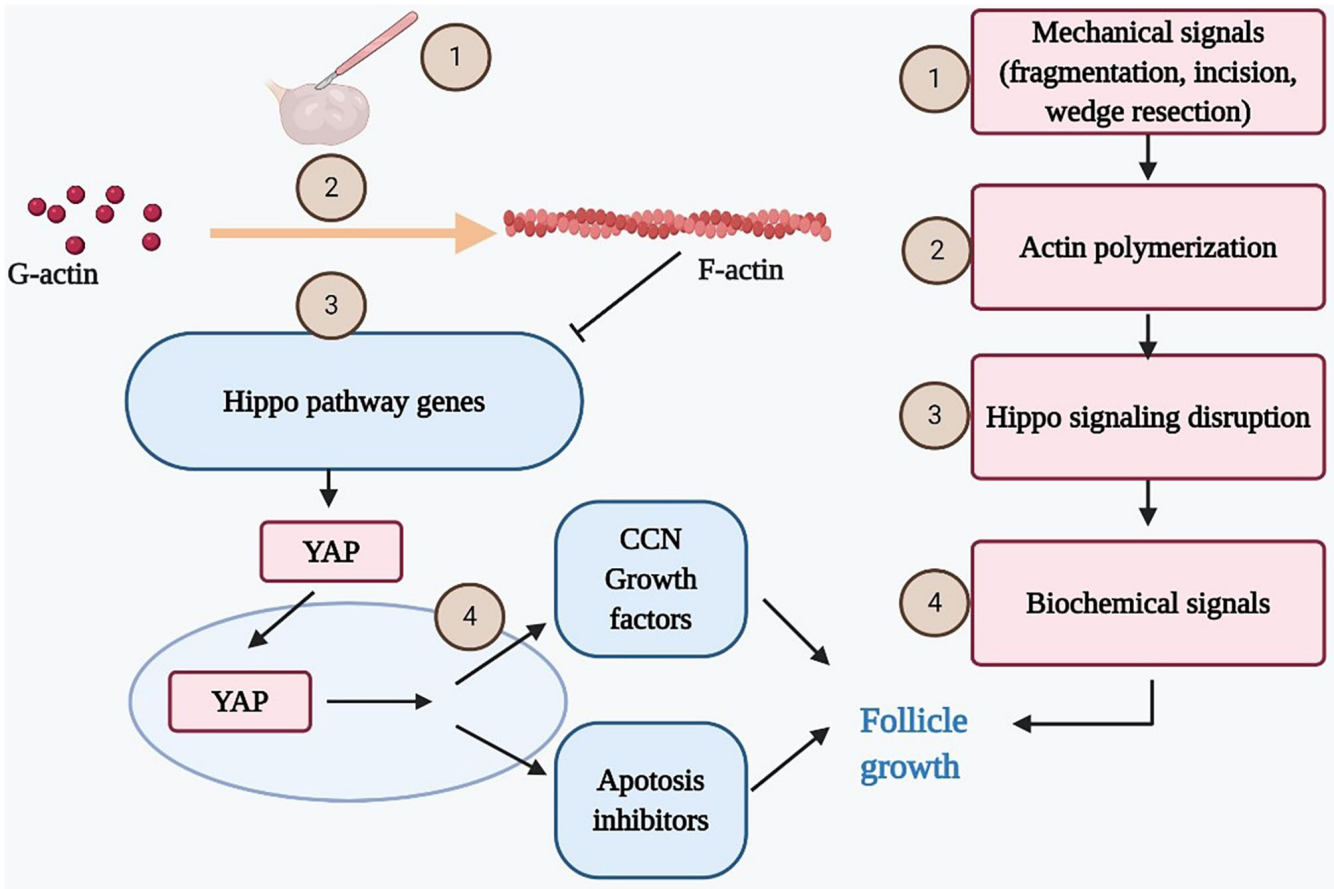
Mechanical force disrupts ovarian Hippo signaling pathway and promotes follicle growth. Mechanical signals incurred by ovarian fragmentation, incision, drilling, or wedge resection lead to actin polymerization that disrupted ovarian Hippo signaling, resulting in nuclear translocation of Yes-associated protein (YAP). Nuclear YAP interacted with transcriptional enhanced associate domain (TEAD) transcriptional factors to increase the expression of downstream biochemical signals (cystein-rich 61, connective tissue growth factor, and nephroblastoma overexpressed [CCN] growth factors and BIRC apoptosis inhibitors), resulting in follicle growth. Ovarian damage–induced follicle growth started with mechanical stimulation but ended with local biochemical changes to promote follicle growth.

In the mammalian ovary, the fragmentation of ovary cortex into small cubes was revealed to disrupt the Hippo signaling pathway by increasing the polymerization of G-actin into F-actin. Consequently, increases in YAP nuclear translocation stimulated the expression of CCN growth factors and BIRC apoptosis inhibitors, resulting eventually in follicle growth (5, 7, 44) (Figure 2). Furthermore, other studies confirmed that the Hippo signaling pathway works in concert with PI3K/Akt activators to accelerate primordial follicle recruitment (6, 45, 46).

Using the animal model, several studies demonstrated thoroughly the mechanism of this intracellular signaling pathway. One hour after fragmentation of ovarian cortex from 10-day-aged mice, the ratios of F-actin to G-actin increased transiently and the decrease of pYAP to total YAP ratios were detected, indicating disruption of the Hippo pathway. The CCN2 transcripts in somatic cells were found to increase using real-time RT-PCR. The ability of CCN proteins in the promotion of follicle growth was also demonstrated based on a dose-dependent increase in ovarian explant weights after culturing with CNN2, 3, 5, or 6. On the 5th day after grafting under hosts' kidney capsules, a remarkable difference in size was noted between the treated ovaries compared to the paired intact ones. In the histology results, there was an obvious increase in percentages of late secondary and antral/preovulatory follicles, along with the decline in early secondary/primordial follicles. After triggering by an ovulating dose of human chorionic gonadotropin (hCG), fragmented grafts had a 3.1-fold higher number of retrievable oocytes compared to intact grafts (7). The development of early embryos from fertilized mature oocytes retrieved from fragmented grafts was comparable to controls. After ET, healthy pups were delivered (7). An animal study demonstrated that promoting the conversion of G-actin to F-actin by jasplakinolide (JASP) or sphingosine-1-phosphate (S1P) in ovaries of 10-day-old CD-1 mice also increased nuclear YAP and expression of downstream CCN2, leading to the enhancement of follicle growth *in vitro* and *in vivo* (44). Besides, the addition of S1P to the culture medium also decreased the follicle atresia and improved the primordial follicle quality (47–49). Similar to the ovarian fragmentation approach, the enzymes degrading the extracellular matrix secreted by granulosa cells were proposed to activate the primordial follicles (50). In contrast, a recent study revealed that S1P treatment could neither activate the primordial follicle nor induce the follicle growth in both mice and human ovaries though the CCN2 gene expression was increased. However, the authors admitted that the longer renewal interval of S1P as compared to one in the study of Cheng et al. (24 vs. 12 h, respectively) could affect the result because the half-life of S1P is as short as 15 min (51). Genetic studies illustrated the importance of Hippo signaling pathway in regulating folliculogenesis. In mice model, a study indicated that YAP is dispensable for oocyte survival, growth, and maturation (52). In humans, deletion of suppressing actin depolymerization genes as well as other related Hippo pathway genes was identified in subfertile or fertile women (53–57).

## The Implementation of Ovarian Fragmentation and AKT Stimulation in Infertility Treatment For POI Patients

POI, characterized by early exhaustion of ovarian function, affecting 1–2% of the population (58, 59). Oocyte donation is currently the popular option for infertility treatment in POI patients. However, several considerations need to be addressed with oocyte donation. The principal concern is the fact that patients cannot have their genetically related offspring, leading to personal and ethical issues. In some countries, oocyte donation is prohibited due to ethical issues and religious reasons (e.g., many Islamic countries). According to the survey of the International Federation of Infertility Society, 41 out of 215 countries do not allow oocyte donation (60). Moreover, some papers reported that oocyte donation resulted in high-risk pregnancies due to immune compatibility (61, 62). A recent meta-analysis concluded that oocyte donation is related to an increased risk of preeclampsia in singleton pregnancies (63).

**Figure 3:**
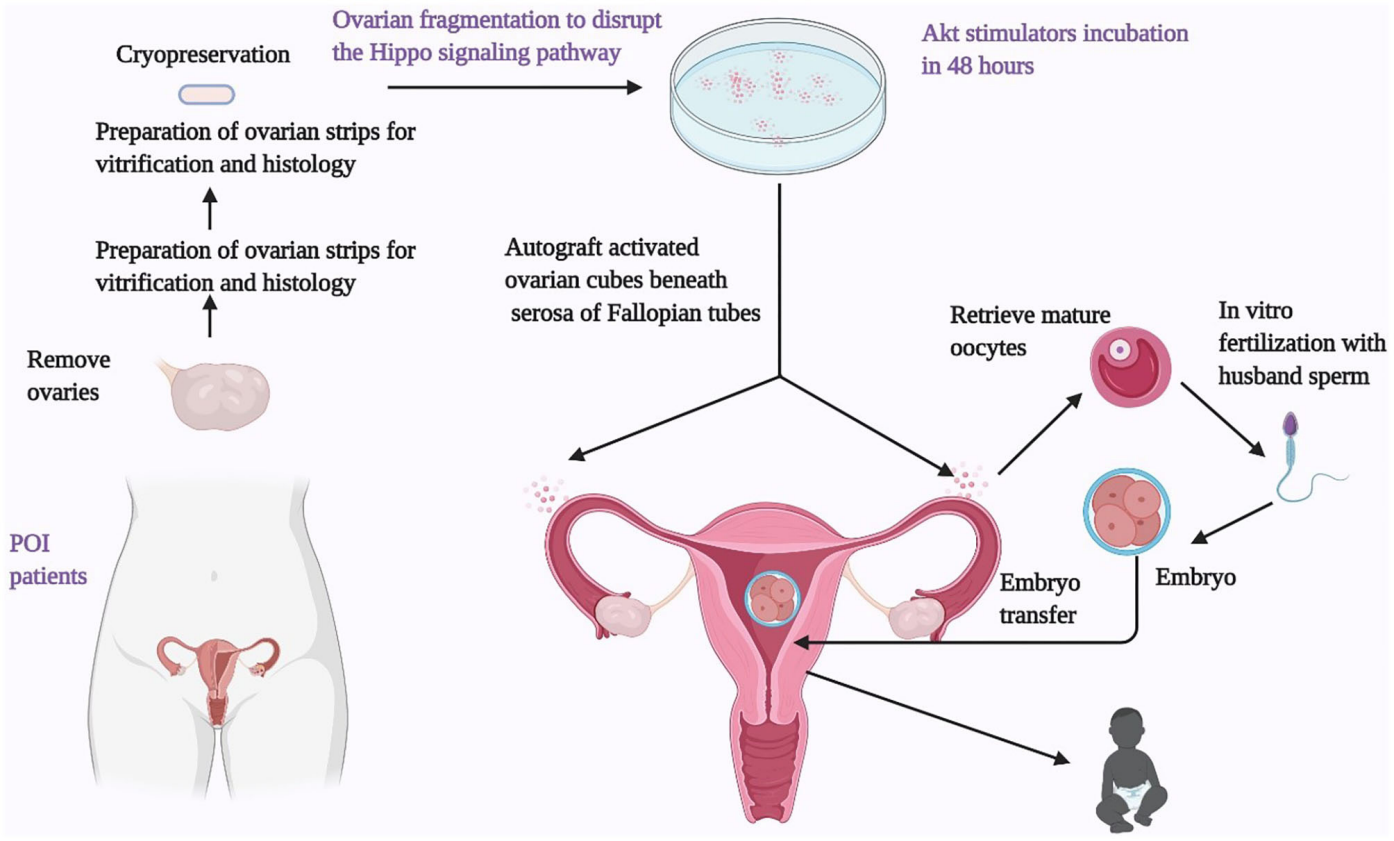
Ovarian fragmentation/AKT stimulation followed by autografting promotes follicle growth in POI patients to generate mature oocytes for IVF embryo transfer, pregnancy, and delivery. Under laparoscopic surgery, one or both ovaries from POI patients were removed and cut into strips before vitrification. After thawing, strips were fragmented into 1–2 mm^2^ mm cubes, before incubation with AKT stimulators (a PTEN inhibitors and a PI3K stimulator). Two days later, cubes were autografted under laparoscopic surgery beneath the serosa of Fallopian tubes. Follicle growth was monitored weekly or biweekly *via* transvaginal ultrasound and based on serum estrogen levels. After detection of antral follicles, patients were treated with FSH followed by human chorionic gonadotropin when preovulatory follicles were found. Mature oocytes were then retrieved and fertilized with husbands' sperm *in vitro* before cyropreservation of 4-cell-stage embryos. Patients then received hormonal treatments to prepare the endometrium for implantation followed by transferring of thawed embryos.

Given the current knowledge of the PI3K/Akt/FOXO3 and Hippo pathways in follicle growth, IVA has been recently introduced to treat POI women. In this approach, the ovarian cortices are fragmented into small cubes (1–2 mm) followed by *in vitro* culture with a PI3K stimulator and a PTEN inhibitor for 2 days and grafting beneath the serosa of the fallopian tubes (Figure 3). Several clinical studies have reported the effectiveness and safety of this treatment. The first pregnancy of this procedure was reported in 27 POI patients with 37.3 ± 5.8 years of age, and a long duration of amenorrhea (6.8 ± 2.1 years). Under laparoscopic surgery, ovaries were removed and cut into strips (1 × 1 cm with 1–2 mm thickness) before vitrification. Following histological analyses, ovaries from 13 out of 27 patients were found to contain residual follicles. Frozen ovarian strips were thawed and fragmented into ~100 cubes (1 × 1 × 1 mm), followed by the incubation with Akt stimulating drugs in 2 days. The ovarian cortical cubes were subsequently transplanted beneath the serosa of the fallopian tubes. Following weekly or biweekly transvaginal ultrasound monitoring under ovarian stimulation, follicle growth was found in eight patients. In five patients, mature oocytes were successfully retrieved for intracytoplasmic sperm injection (ICSI) using the husband's sperm. A healthy male baby was delivered at term with normal physical features (7). A subsequent study using the same procedure was conducted on 37 POI patients. 54% (20/37) of these patients were found to have residual follicles based on histology. Nine out of these 20 women had follicle growth, leading to 24 retrieved oocytes in six patients. After IVF-ET in four patients, three clinical pregnancies were detected, followed by one miscarriage and two healthy live births (11). Another study performed IVA treatment in 14 POI patients with a mean duration since the last menses of 3.8 years. Eleven of the 14 patients had undetectable AMH levels. During the 1-year follow-up period after the IVA procedure, a total of 15 follicle developmental waves were detected in six of the 14 patients (43%), resulting in six oocytes from four patients. After IVF-ET of retrieved oocytes, one patient delivered a healthy baby boy, with three other frozen embryos (12).

Furthermore, drug-free IVA, the simplified procedure of IVA, was also demonstrated to be successful in treating POI patients at the early stage. In this approach, ovarian cortex is fragmented to disrupt ovarian Hippo signaling followed by the grafting back into remaining ovaries and beneath serosa of Fallopian tubes without tissue culture. Several studies reported successful outcomes by using the drug-free IVA procedure. A case report from Spanish group represented that a 32-year old patient with elevated FSH levels (89.9 mIU/mL) and undetectable AMH levels (<0.02 ng/ml) became pregnant after the drug-free IVA procedure followed by 20 days of ovarian stimulation (13). Another study from the same group carried out in 14 POI women demonstrated the positive outcome of drug-free IVA. These patients were at the early stage of POI with median age of 33 years (29–36 years), a median length of amenorrhoea of 1.5 years (1–11 years), and a median AMH level of 0.02 ng/ml (0.01–0.1 ng/ml). After the drug–free IVA procedure, follicle development was detected in seven patients, and five women achieved successful oocyte retrieval. These five women had six embryo transfers, resulting in four pregnancies (14). Furthermore, a large trial from Chinese group described the promising outcome of the disruption of Hippo signaling pathway by ovarian biopsy and scratching in 80 POI women. Eleven (13.75%) patients presented with spontaneously ovarian function resumption or follicle growth after human menopausal gonadotropin stimulation. Ten patients underwent oocyte retrieval and IVF, two embryos were transferred to one patient, and one healthy baby was delivered (64).

Although POI patients can achieve pregnancy spontaneously or by other ART therapies (65), these above data demonstrated that IVA yielded better infertility treatment outcomes for POI patients. In a cohort study enrolling 358 young POI patients (mean age: 26.6 ± 7.9 years), the spontaneous pregnancy rate was 4.4% during 13 years (66). In other studies conducted in young POI patients (median age: 30.36–32.5 years of age), the pregnancy rates ranged from 3.6 to 6.8% after hormonal therapy with or without ART (67–70). In the second report of IVA study (11), among 37 older POI patients (37.6 ± 4.6 years of age), twenty patients had residual follicles by the histological analysis, 15.0% (3/20) of patients conceived after the IVA procedure within 1 year and two more patients had cryopreserved embryos pending ET (11). However, these included studies are case series with a limited number of patients, lacking the control group with sham operation. Moreover, long-term neonatal safety is still limitedly examined by genetics examinations. Additional investigations are essential to confirm these findings and ensure the safety of this approach.

A recent study challenged the effectiveness of IVA by xenografting human ovarian cortical tissues to immunodeficient mice. In this study, ovarian tissues from 18 young women were divided into three groups (fresh, slow-frozen, and vitrified). These cortical tissues were fragmented into small cubes followed by with or without culture in Akt stimulators, before xenografting to the peritoneal pockets. The investigators concluded that ovarian fragmentation and Akt stimulation yielded no significant benefits in terms of growing follicle percentages or follicle proliferation rates (71).

However, this study described the follicle density (follicles per mm^2^) which is not highly valid to investigate the benefit of IVA. Since the fundamental mechanism of Hippo pathways and Akt stimulation is improving the development of the primordial follicle with a smaller size to the later stage follicles with a larger size, the follicles density is supposed to not be higher (but even lower) after grafting. Indeed, a significant decrease in follicle density after transplantation was found in this study (71). In terms of the percentage of growing follicles, the duration of 28 days after xenografting is fairly short for evaluating the transition of the primordial follicles to the later stages. Furthermore, the peritoneal pockets as the grafting site may supply a lower blood stream compared to the site under kidney capsule with high vascularization (72). This can have negative impacts on follicle growth.

## Perspectives of Hippo Signaling Pathway and AKT Stimulation for Extending Fertile Duration

### POR With DOR

Women have been progressively delaying their childbirth until the third and fourth decades of life, especially in Western countries (2, 3, 73). This leads to POR with DOR, a natural depletion of oocyte quantity as well as decreases in egg quality, representing unsolved problems in reproductive medicine. The successful outcome of IVA approach in POI patients suggested that this treatment was also beneficial for POR patients with advanced age and severe ovarian dysfunction. Since the activation of primordial follicles could happen spontaneously in DOR condition, the drug-free IVA procedure likely promoted secondary follicle growth. A recent case series study in 11 POR with DOR women at advanced age (30–45) and a median AMH level of 0.04 (0–0.8) reported that this procedure increased the number of antral follicles following FSH treatment and the number of mature retrieved oocytes per cycle. The fertilization rate and high quality embryo rate were 68.7 and 56.9%, respectively. In consequence, five patients achieved pregnancies, resulting in one live birth, two ongoing pregnancies, and one miscarriage. Moreover, three patients and the miscarriage patient could have cryopreserved embryos (15). Another case series study reported that 13 out of 15 POI and POR with DOR patients who were treated with the drug-free IVA achieved a higher number of antral follicle numbers as well as a higher number of retrieved oocytes as compared to previous IVF outcomes before the IVA treatment. One spontaneous pregnancy and embryo transfer allowed four live births and one ongoing pregnancy. Five additional patients and one miscarriage patient have cryopreserved embryos for future transfer (16).

On the other hand, one report recently from Denmark raised questions regarding the effectiveness of IVA. Firstly, they conducted a similar drug-free IVA in 20 POI patients. There were no recorded complications and 12 patients could achieve pregnancies (74, 75). There was no significant difference in the number of mature follicles and the AMH levels between the treated group and the control one during 10 weeks of observation (75). Despite high pregnancy rate (60%) after IVA, an editorial suggested the ovarian fragmentation should be eliminated to treat for DOR patients (76). However, 10 weeks was supposed to be rather short duration for monitoring follicle growth and serum AMH levels (9). In this study, seven out of 20 patients presented an increase in AMH level and antral follicle counts, indicating the effectiveness of ovarian fragmentation. It is important to note that our earlier publication indicated that serum AMH levels remained undetectable after IVA treatment when only few follicles reached the preovulatory stage (12).

### Fertility Preservation

As the cancer survival rate among young women has significantly increased recently, efforts to preserve fertility have received significant attention (77). Fertility preservation (FP) by ovarian cryopreservation and autologous transplantation has been practiced in the last two decades and resulted in more than 130 healthy children worldwide (78, 79). Because sectioning is essential during the ovarian tissue cryopreservation, *in vitro* fragmentation and IVA drug treatment can be introduced during the ovarian cryopreservation to enhance the outcome of FP. A study from a Spanish group using 18 human ovarian cortex biopsies from cancer women demonstrated that short term incubation with PTEN inhibitor enhanced the development of growing follicles as well as the surrounding stroma populations without inducing apoptosis. The AMH concentration in the fresh activated samples was significantly higher compared to the control group (35). In consistence, a recently published study from a Belgium group indicated that fragmentation increased the number of secondary follicles in oncological patients (80).

The ovarian tissue cryopreservation and IVA approach are also likely beneficial to other populations, including women about to be treated with gonadotoxic agents as well as for women with other non-malignant diseases including endometriosis or immune disorders (79, 81). Additionally, FP is a favorable option for either unmarried women with severe ovarian dysfunction or women wishing to postpone childbearing for various personal reasons. Of note, some studies reported that the activation of primordial follicle could occur spontaneously after transplantation of frozen-thawed ovarian tissues using the conventional cryopreservation method (82–84) and transient incubation with mTOR inhibitors extended the graft lifespan by preventing the massive activation (45, 85). Although the IVA procedure can activate more dormant primordial follicles in frozen-thawed ovarian samples (28), others raised the concern about ovarian endocrine function and reproduction capacity after IVA at long-term goal since the conventional IVA approach provokes an immediate follicular activation (86).

### Resistant Ovary Syndrome and Polycystic Ovarian Syndrome

In addition to the aforementioned perspectives, the application of Hippo signaling pathway can be beneficial to other ovulatory disorders. In detail, some women represent with ovaries unresponsive to endogenous and exogenous gonadotropins, in spite of normal ovarian reserve. This condition has been referred to as ROS, a rare disorder that could not be treated with routine ovarian stimulation (87). Based on the successful outcomes after *in vitro* fragmentation in POI and DOR patients, it is hypothesized that the incision of ovarian cortex *in vivo* to disrupt Hippo pathway can stimulate arrested follicles in ROS patients. Interestingly, ovarian incision through laparoscopic surgery was found to promote follicle growth and yield successful oocyte retrieval in seven of 11 ROS patients (16). Although this approach could be a better option compared to IVA, there are no published studies comparing the clinical outcome between IVA and ovarian incision in patients with ovarian dysfunction. Future comparative studies might develop a more efficient and less invasive treatment.

Another ovulatory disorder is polycystic ovarian syndrome (PCOS), the common endocrinopathy affecting approximately 8.7–17% of women in the reproductive age group (88). Although PCOS patients could achieve pregnancies through current ART practice, some PCOS women faced multiple challenges including poor to an exaggerated response, poor oocyte quality, poor fertilization rate, poor blastocyst conversion, and ovarian hyperstimulation syndrome (88). Ovarian wedge resection and ovarian drilling have been shown to induce follicle growth in PCOS patients, especially in clomiphene citrate-resistant cases (89, 90), suggesting induction of follicle growth by alterations in mechanical tensions. There have been several studies that indicated the association between the expression of genes related to Hippo pathway and PCOS condition (91–93). Besides, hypomethylation of the YAP promoter was found to be a key pathogenesis of PCOS (94). Consequently, it is logical to suppose that Hippo signaling pathway is correlated with PCOS. Hippo gene-targeted therapeutics would be effective on fertility and systemic symptoms of PCOS (91). The incision of ovarian cortex or the use of pharmacologic agents targeting this pathway could be applied to PCOS patients to normalize follicle growth and ovulation while minimizing the damage to ovarian reserve. Further studies are necessary to evaluate the safety and efficacy of the ovarian incision in PCOS patients.

## Conclusion

In conclusion, social changes and the increasing desire for parenthood of infertile couples have increased the range of ART. The implementation of ovarian fragmentation and Akt stimulators can increase the chance to conceive genetically related children for various types of poor prognostic infertile women, leading to the expansion of modern infertility treatment. However, the discussed studies involve a small group of patients, further confirmation by better designed studies is essential for the wide clinical implementation of IVA therapy. The necessity of preparation of a control group with sham operation of IVA makes it difficult and ethically unjustified. Besides, it is necessary to develop a less invasive method to predict the presence of residual follicles before ovariectomy as well as an alternative approach to disrupt Hippo signaling pathway (e.g., actin polymerization-enhancing reagents) to improve the efficiency of IVA.

## Author Contributions

KK: designed the outline of review. KV and KK: wrote the manuscript.

## Conflict of Interest

The authors declare that the research was conducted in the absence of any commercial or financial relationships that could be construed as a potential conflict of interest.
